# Ultra high content analyses of circulating and tumor associated hybrid cells reveal phenotypic heterogeneity

**DOI:** 10.1038/s41598-024-57381-8

**Published:** 2024-03-28

**Authors:** Riley M. Whalen, Ashley N. Anderson, Jocelyn A. Jones, Zachary Sims, Young Hwan Chang, Michel A. Nederlof, Melissa H. Wong, Summer L. Gibbs

**Affiliations:** 1 Department of Cell, Developmental, and Cancer Biology, Oregon Health & Science University (OHSU), Portland, OR 97201 USA; 2 Department of Biomedical Engineering, OHSU, Portland, OR 97201 USA; 3 Knight Cancer Institute, OHSU, Portland, OR 97201 USA; 4 Quantitative Imaging Systems, LLC, Pittsburgh, PA 15238 USA

**Keywords:** Cyclic immunofluorescence, Circulating hybrid cell, Cancer biomarker, Oligonucleotide-conjugated antibody, Cancer progression, Colorectal cancer, Pancreatic cancer, Cancer, Cell biology

## Abstract

Persistently high, worldwide mortality from cancer highlights the unresolved challenges of disease surveillance and detection that impact survival. Development of a non-invasive, blood-based biomarker would transform survival from cancer. We demonstrate the functionality of ultra-high content analyses of a newly identified population of tumor cells that are hybrids between neoplastic and immune cells in patient matched tumor and peripheral blood specimens. Using oligonucleotide conjugated antibodies (Ab-oligo) permitting cyclic immunofluorescence (cyCIF), we present analyses of phenotypes among tumor and peripheral blood hybrid cells. Interestingly, the majority of circulating hybrid cell (CHC) subpopulations were not identified in tumor-associated hybrids. These results highlight the efficacy of ultra-high content phenotypic analyses using Ab-oligo based cyCIF applied to both tumor and peripheral blood specimens. The combination of a multiplex phenotypic profiling platform that is gentle enough to analyze blood to detect and evaluate disseminated tumor cells represents a novel approach to exploring novel tumor biology and potential utility for developing the population as a blood-based biomarker in cancer.

## Introduction

Over 18 million new cases of cancer are diagnosed annually worldwide, resulting in over 10 million deaths. These staggering statistics highlight the challenges of management and surveillance of cancer, which include effective early detection, monitoring of disease burden, and identification of tumor recurrence after treatment. These challenges are especially relevant in pancreatic ductal adenocarcinoma (PDAC) and colorectal cancer (CRC), as almost half of the patients with CRC recur with incurable disease^1,2^ and the majority of PDAC patients present with late-stage, treatment-refractory disease due in part to disease heterogeneity^3^. These challenges, and thus overall cancer mortality, could be addressed with the development of effective, non-invasive biomarkers to provide longitudinal surveillance of disease burden and tumor response to treatment.

Current modalities of detection and monitoring include PET/CT imaging^4^, as well as molecular and pathologic analyses of tumor biopsies. Many barriers to these approaches exist, including detection sensitivity and cost of imaging, and the highly invasive nature of tumor biopsies which pose increased risks to the patient^5^. Further, longitudinal biopsies are not always an option after primary tumor resection. In addition, the information gained from analyses of a tumor biopsy does not necessarily represent the information and landscape of the entire tumor, as it consists of only one small region^1^. Thus, there is an unmet need for a tumor biomarker that is non-invasive, repeatable, and comprehensive.

The development of blood-based biomarkers that reflect tumor burden and biology is an important advancement in tumor monitoring and disease control. Notably, a peripheral blood draw is less invasive and can be acquired multiple times. Moreover, a myriad of cancer blood-based biomarkers are in development and demonstrate strong concordance with primary tumor attributes, including proteins, exosomes, cell-free nucleotides, and circulating cells^1,5–8^. Of these biomarkers, disseminated cells provide the most versatility allowing for analyses of cell state and disease burden because they are directly derived from the tumor, secrete exosomes, and harbor nucleic acids (RNA and DNA) and proteins. Of disseminated tumor cells, circulating tumor cells (CTCs)^8^ are the most developed cell-based cancer biomarker with FDA-approved assays, however, these cells are rare in circulation (i.e., 1–5 CTCs in 7.5 ml of blood correlates with high cancer burden)^9–12^. Despite their rarity, CTCs are reported to display phenotypic heterogeneity with described subpopulations harboring epithelial to mesenchymal (EMT) signatures. However, the analyses of these subpopulations have not demonstrated their value in predicting tumor progression or patient outcomes^13,14^. Our group identified a disseminated neoplastic cell that can derive from cell fusion between a cancer cell and an immune cell (commonly a macrophage), which is called a cancer-immune hybrid cell^15,16^. These recently identified hybrid cells retain malignant cancer cell characteristics and harbor immune cell features of increased mobility^15,16^. Tumor-immune hybrid cells are distinguished from cancer-associated macrophage-like cells (CAMLs) and immune cells that have phagocytosed dying cancer cells because they have cell surface antigen expression of both cancer and immune cell epitopes^5^. Additionally, when disseminated into peripheral blood, circulating hybrid cells (CHCs) are more abundant than CTCs, and have greater metastatic seeding capabilities^15–17^. Moreover, CHCs display concordant protein expression and DNA mutations with the primary tumor^15–18^. CHCs are detected in patient blood across cancer types and disease stages^17–27^ and their increased numbers correlate with poor patient outcomes and greater disease burden after treatment in PDAC and CRC, respectively^15,18^. Thus, to enhance the ability of CHCs to provide biologic insight for tumor progression and clinical information for disease burden, we developed an approach to deeply phenotype hybrid cells within a tumor section and in corresponding peripheral blood.

Herein, we implement our previously published, optimized oligonucleotide conjugated antibody (Ab-oligo) strategy enabling our cyclic immunofluorescence (cyCIF) platform^28–30^ to interrogate protein expression in tissue sections and in corresponding peripheral blood. This Ab-oligo cyCIF platform improves upon aspects of other multiplex techniques, including gentle signal removal between rounds of antibody staining, which is crucial for analyses of peripheral blood specimens. Specifically, Ab-oligo cyCIF employs single-stranded DNA conjugated to antibodies, which is detected by in situ hybridization to complementary DNA strands with photocleavable linkers (PCLs) that are conjugated to fluorophores. In between rounds of Ab-oligo staining and after imaging, the fluorescence signal is removed using ultraviolet (UV) light treatment. Like traditional immunofluorescence (IF) and immunohistochemical (IHC) staining techniques, including other cyclic techniques^31^, our Ab-oligo cyCIF strategy permits intracellular and single-cell resolution of multiple targets per cell^28–30^. Additionally, by employing UV-cleavable DNA barcodes, signal removal is achieved without compromising antigenicity or sample integrity. Unlike other DNA-barcoding techniques^32–38^, which often require specialized equipment, the Ab-oligo cyCIF staining protocols^1^ are similar to other IF approaches and thus are easily adopted. Herein, we demonstrate the application of the Ab-oligo cyCIF multiplex protocol for rapid and specific protein detection in CRC and PDAC patient tumor tissues and peripheral blood samples acquired at the time of resection in order to investigate phenotypic concordance between tumor-associated hybrids and disseminated CHCs. Analyses using this approach will unravel discrete phenotypic signatures that underly the biology of metastatic dissemination of neoplastic cells and provide insights into risk of metastatic progression in patients with cancer.

## Results

**Figure 1:**
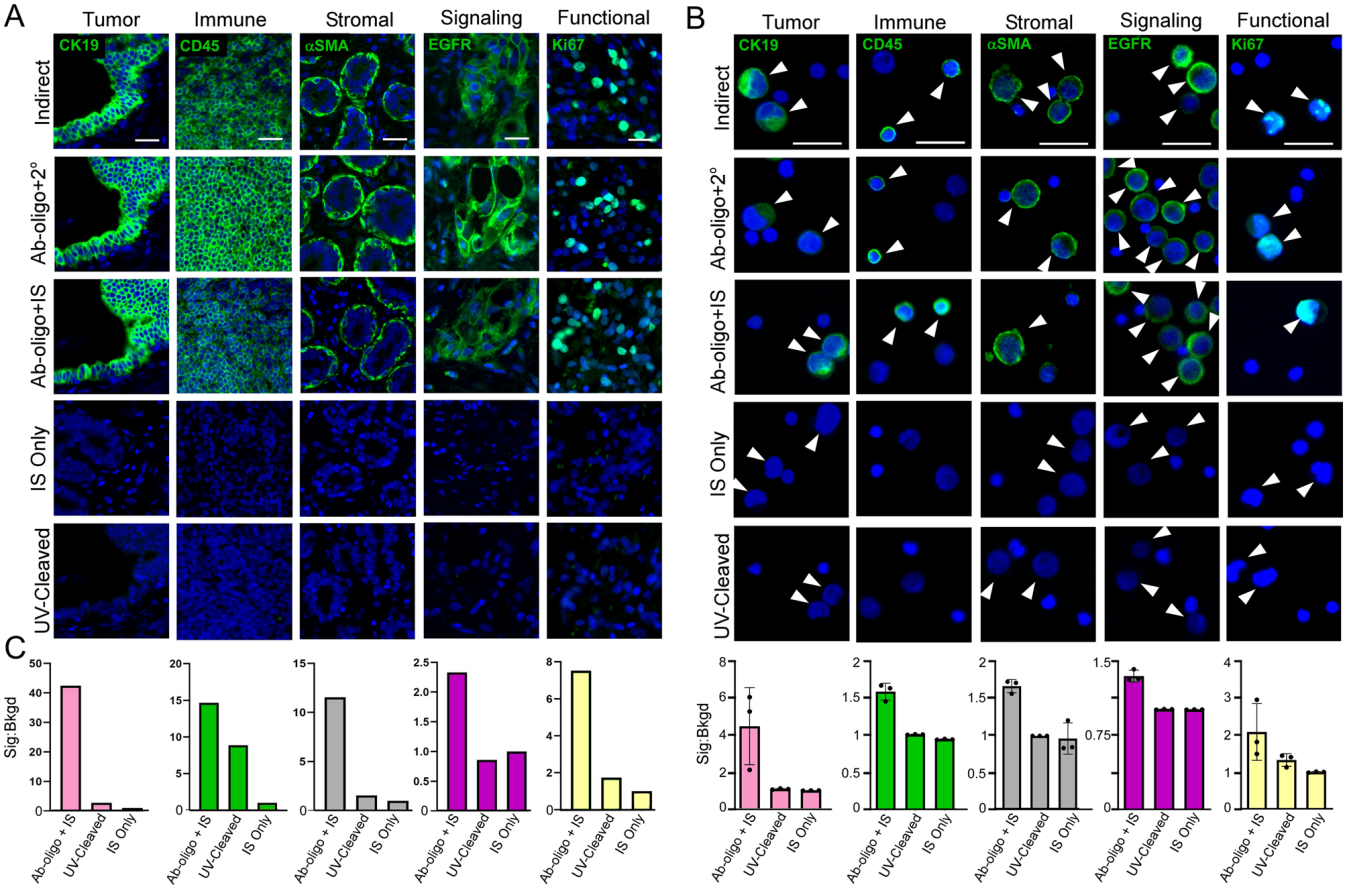
Ab-oligo validation of representative biomarker from five phenotypic categories: tumor, immune, stromal, cell signaling, and functional. (**A**) Formalin-fixed paraffin embedded (FFPE) 5 µm tissue sections, and (**B**) a model of peripheral blood (cancer cell line mixed with peripheral blood mononuclear cells, PBMCs). Antibody or control staining pattern (green) and DAPI nuclear stain (blue) shown in all panels. Antigen detection was by indirect immunofluorescence (primary + secondary antibody, 2°), Ab-oligo plus secondary antibody, and Ab-oligo plus imaging strand (IS). Control settings included UV-cleavage after Ab-oligo + IS staining, and IS only. (**B**) White arrowheads designate staining on cells with antibody, or no staining on controls. (**C**) Graph of signal to background ratio for tissue and peripheral blood model. Ab-oligo + IS, UV-cleaved, and IS only were normalized to the IS only control in stained tumor and to the negative cell population within each validation condition for the blood model. The signal to background ratio was above 1 for all markers. Scale bar = 20 µm. αSMA, alpha smooth muscle actin; Ab-oligo, oligo conjugated antibody; *CK* cytokeratin, *EGFR* epidermal growth factor receptor, *IS* imaging strand, *Sig:Bkgd* signal to background ratio, *UV* ultraviolet.

Co-expression of cancer (i.e., neoplastic) and immune epitopes define cancer-immune hybrid cells within tumors and when disseminated into peripheral blood^15–18^. To determine if concordant phenotypes exist between tumor-associated hybrids and circulating hybrids, we first identified then deeply phenotyped hybrids in patient-match tumor tissue sections and peripheral blood samples collected at the time of tumor resection. We leveraged cyCIF, which allows for multiple rounds of antigen staining and imaging with gentle signal removal between rounds that we demonstrated to be compatible with peripheral blood analyses^30^. To establish our hybrid cell phenotyping Ab-oligo panel we selected antigens from five discrete categories: tumor expression, immune cell, stromal, cell signaling, and functional (Table S1) to allow for analyses of hybrid cell features (i.e., proliferative, cell signaling status, phenotypic/functional) relative to discrete tumor landmarks (i.e., stromal, immune). After generation of corresponding Ab-oligos, we demonstrated that each Ab-oligo (i.e., Ab-oligo + Imaging Strand, IS) had consistent staining patterns in tumor tissue relative to conventional indirect antibody staining (i.e., using primary antibody with fluorophore conjugated-secondary antibodies) and to Ab-oligo conjugates detected using conventional secondary antibody staining (Fig. 1A, Fig. S1). Further, we evaluated the signal to background ratio of the Ab-oligo + IS versus tissue treated with IS only and also Ab-oligo + IS stained tissue treated with UV light to cleave the fluorophore-conjugated IS signal. In all cases, the specific Ab-oligo signal to background ratio was greater than the controls (i.e., greater than 1) (Fig. 1C). Next, we validated the Ab-oligo staining pattern on a model of peripheral blood—human cancer cell lines spiked into peripheral blood mononuclear cells (PBMCs) isolated from a healthy subject to assess antibody specificity to tumor or immune cells. Consistent with results from tumor tissue staining, Ab-oligos displayed identical staining patterns relative to indirect antibody staining and Ab-oligo + secondary antibodies (Fig. 1B, Fig. S2). Further, the signal to background ratio of the Ab-oligo + IS was greater than the controls (i.e., greater than 1) for all Ab-oligos in the panel, except for VWF which did not stain either cell population.

**Figure 2:**
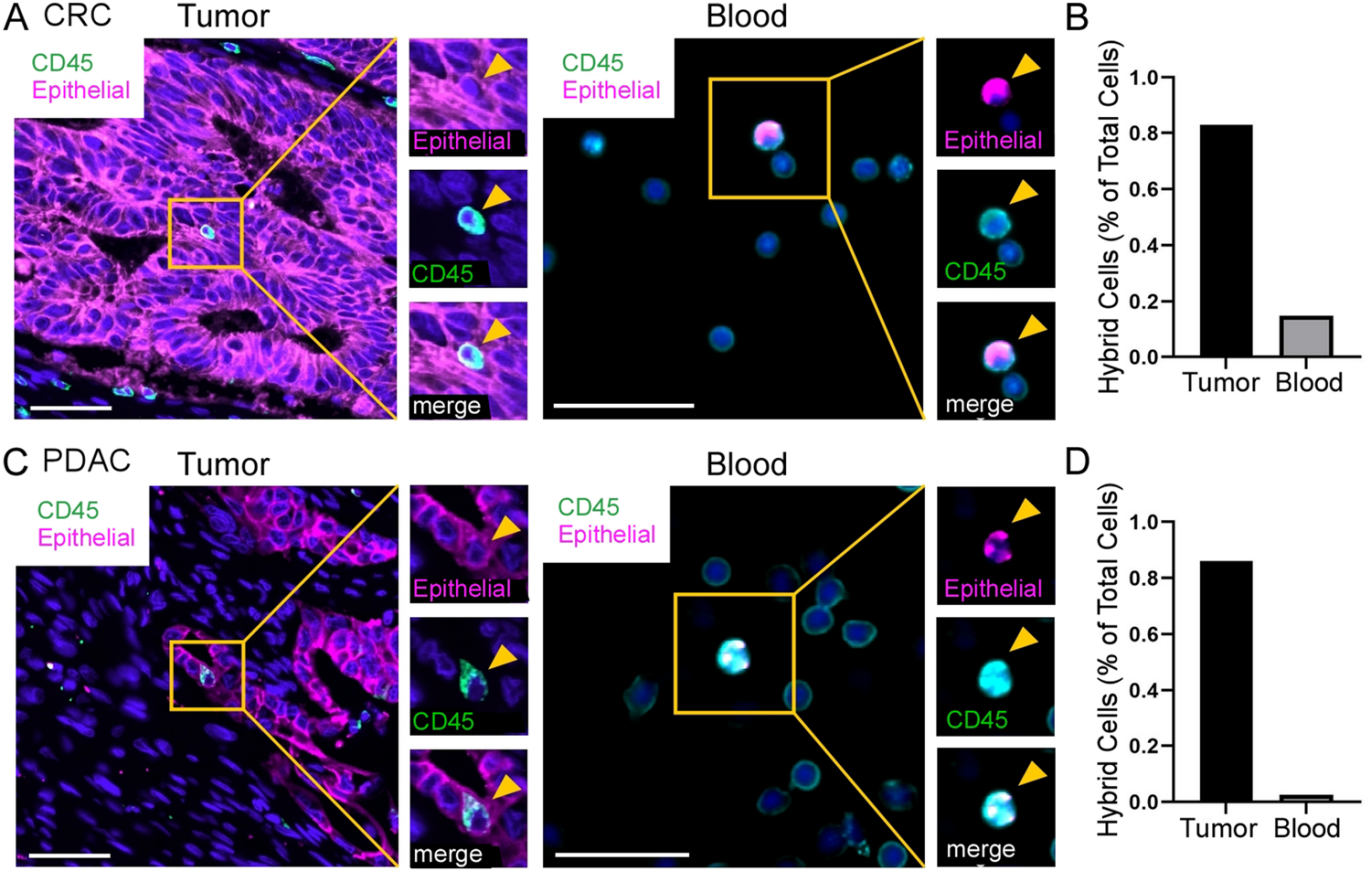
Hybrid cell identification in tumor tissue and peripheral blood. (**A**) Colorectal cancer (CRC) and (**C**) pancreatic ductal adenocarcinoma tumor (PDAC) and peripheral blood specimens stained with oligo-conjugated antibodies (Ab-oligos) to CD45 (green), the epithelial cocktail (Epithelial: ECAD, EpCAM (pink), and panCK) and DAPI (blue). Hybrid cells are boxed in yellow; single channel images are displayed on right with yellow arrowhead marking hybrid cell. (**B**,**D**) Quantification of hybrid cells. Scale bar = 50 (tissue) or 10 (cells) µm. *CRC* colorectal cancer; *PDAC* pancreatic ductal adenocarcinoma.

To identify hybrid cells in patient-matched tumor and peripheral blood, we applied a small panel of Ab-oligos against CD45, a pan-leukocyte marker, and an epithelial cocktail containing Ab-oligos to EpCAM, ECAD, and pan-Cytokeratin (panCK) to CRC and PDAC tumor tissue and matched isolated PBMCs. Using this panel, we identified numerous hybrid cells in primary tumors and peripheral blood for both CRC and PDAC samples (Fig. 2). Hybrid cells in the tumor tissue co-expressed epithelial proteins (pink) and CD45 (green). Likewise, the corresponding CHCs consistently co-expressed proteins from the epithelial cocktail and CD45. Overall in the paired CRC specimens, we identified 1907 hybrids in one 5 µm tumor tissue section (0.83% of total tumor cells analyzed, Fig. 2B), and 237 hybrids in 1.5 mL of peripheral blood (0.15% of total cells analyzed, Fig. 2B). In the paired PDAC specimens, we identified 1973 hybrids in one 5 µm tumor tissue section (0.86% of total tumor cells analyzed, Fig. 2D), and 74 hybrids in 1.5 mL of peripheral blood (0.025% of total cells analyzed, Fig. 2D).

**Figure 3:**
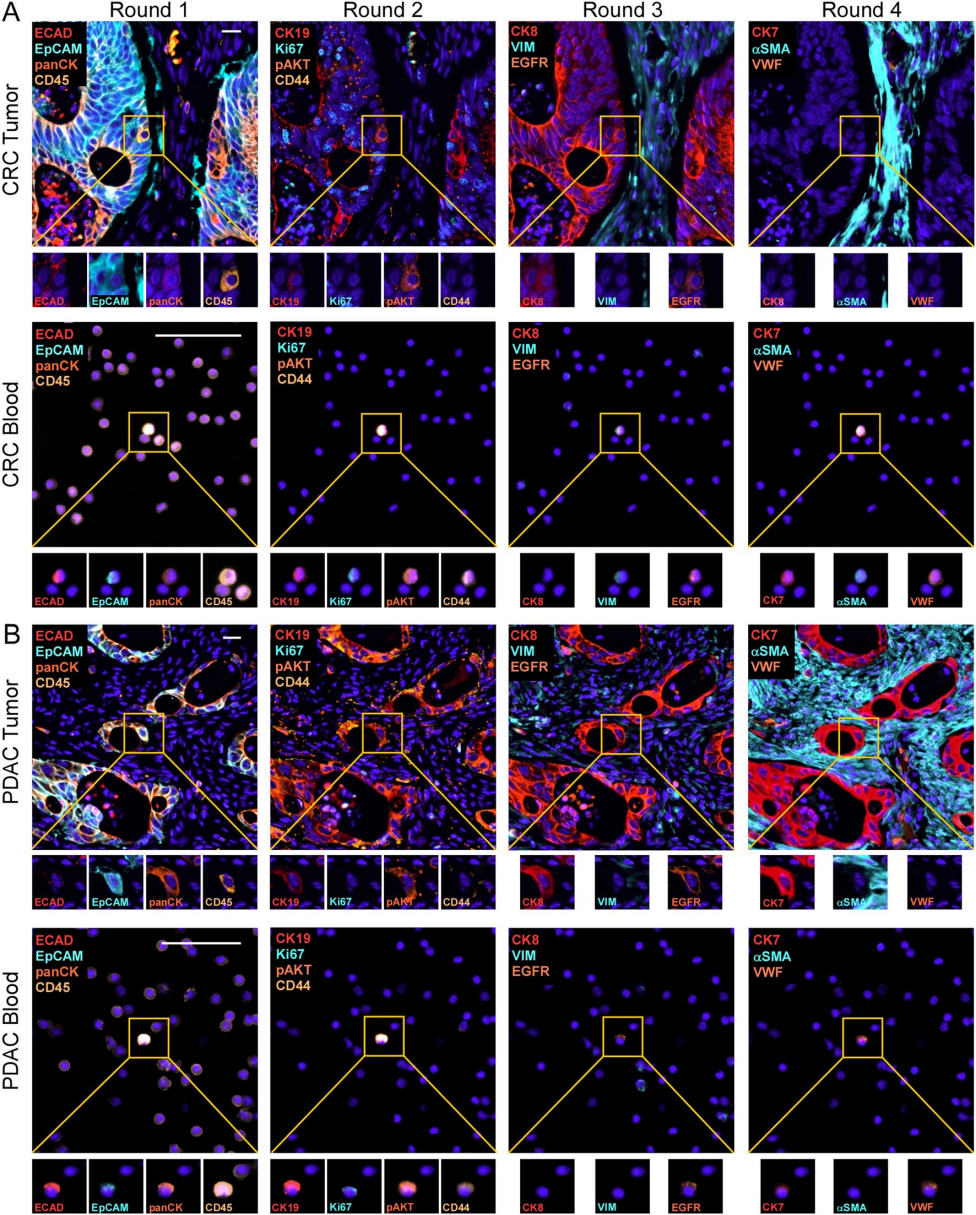
Four rounds of cyCIF to identify and phenotype hybrid cells on patient-matched colorectal cancer (CRC) and pancreatic cancer (PDAC) tumor sections and blood specimens. Each round utilized imaging strands conjugated to Alexa Fluor (AF)546, AF647 and AF750 fluorophores. DAPI nuclear stain was in blue. Round 1: ECAD, EpCAM, panCK, CD45; Round 2: CK19, Ki67, pAKT, CD44; Round 3: CK8, VIM, EGFR; and Round 4: CK7, αSMA, VWF. Hybrid cell in yellow box. Higher magnification, single antigen staining displayed below. Scale bar = 25 µm. αSMA, alpha smooth muscle actin; CK, cytokeratin; CRC, colorectal cancer; ECAD, E-cadherin; EGFR, epidermal growth factor receptor; EpCAM, epithelial cell adhesion molecule; pAKT, phosphorylated protein kinase, strain AK, Thymoma (phosphorylated protein kinase B); panCK, pan-cytokeratin; PDAC, pancreatic ductal adenocarcinoma; VIM, vimentin; VWF, von Willebrand factor.

We next applied our validated panel of Ab-oligos in three additional rounds of staining to the patient matched samples to achieve greater phenotypic information from the hybrid cells. Hybrid cells were identified in the first round with co-expression of CD45 and at least one epithelial marker: ECAD, EpCAM, and/or panCK (Fig. 3, round 1). In between each round of Ab-oligo staining we treated the specimens with UV light and rescanned the tissue to ensure complete erasure of the fluorescent signal before proceeding to the next round of staining. Additional rounds of staining included: round 2 (CK19, Ki67, pAKT, CD44); round 3 (CK8, VIM, EGFR); and round 4 (CK7, αSMA, VWF). In order to superimpose all rounds of staining into a single image, we registered the images using QiTissue, a multiplex image visualization and analysis software package, or feature-based image registration^39^. This allowed us to identify and phenotype hybrid cells within the tumor tissue and corresponding peripheral blood preparation (Fig. 3).

**Figure 4:**
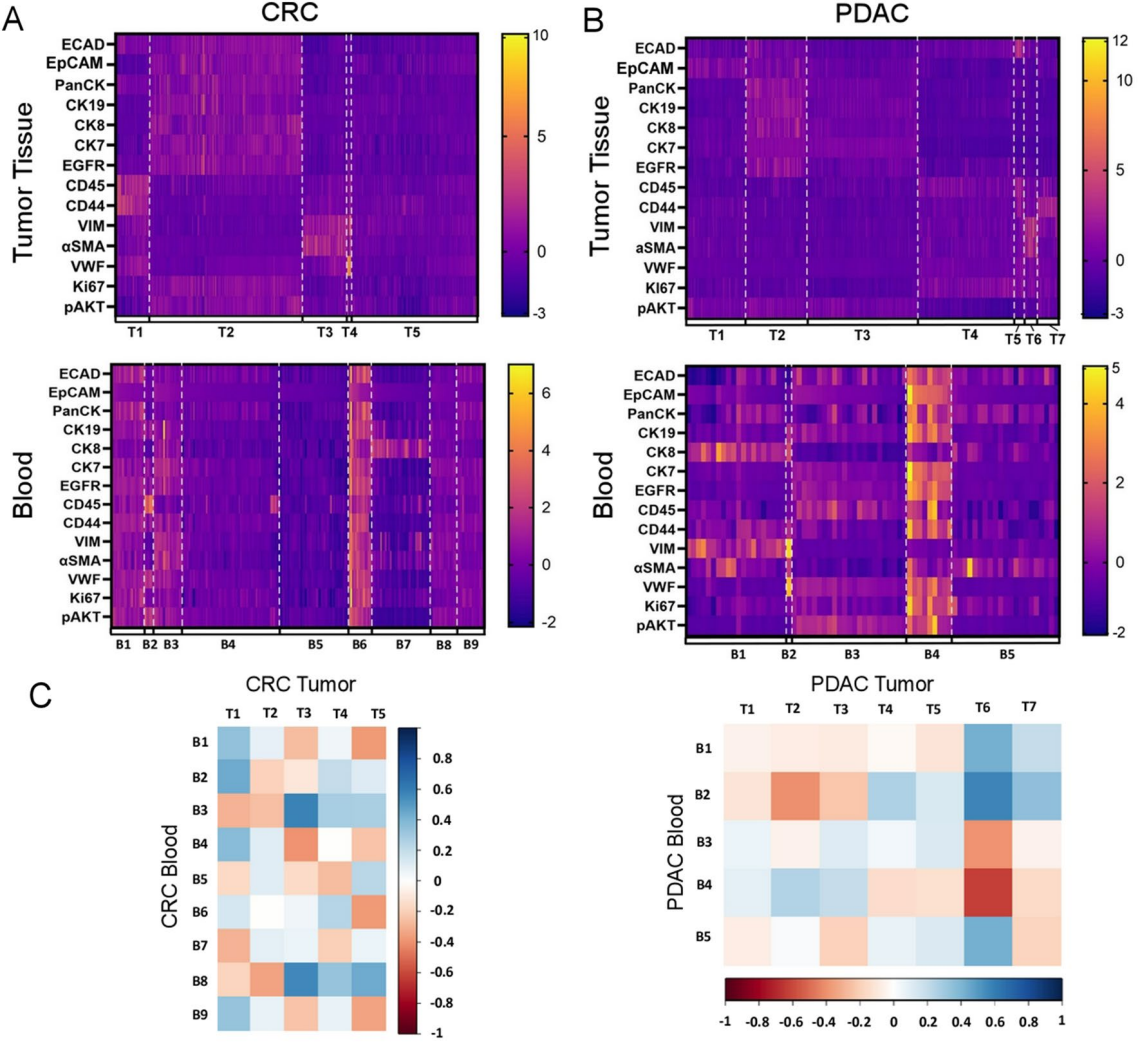
Hybrid cell subpopulations. (**A**, **B**) K-means cluster analysis of normalized fluorescence intensities from identified hybrid cells in colorectal cancer (CRC) and pancreatic cancer (PDAC) tumor sections and peripheral blood depicted as a heat map. Each column represents a single hybrid cell (CD45^+^ and ECAD^+^, EpCAM^+^, or panCK^+^) and each row represents the normalized fluorescent intensity of an antigen in the Ab-oligo panel. Cells were grouped by subpopulation. (**C**) Pairwise correlation analysis between hybrid cell clusters in the tumor section and those in the blood in CRC and *PDAC* patient matched specimens. *αSMA* alpha smooth muscle actin, *CK* cytokeratin; *CRC* colorectal cancer, *ECAD* E-cadherin, *EGFR* epidermal growth factor receptor; *EpCAM* epithelial cell adhesion molecule, *pAKT* phosphorylated protein kinase, strain AK, Thymoma (phosphorylated protein kinase B); *panCK* pan-cytokeratin *PDAC* pancreatic ductal adenocarcinoma, *VIM* vimentin, *VWF* von Willebrand factor.

To directly compare the identified hybrid phenotypes within the tumor tissue to those found disseminated in peripheral blood, we extracted the average fluorescence intensity of each Ab-oligo in our panel from each of the identified hybrid cells in the primary tumor and the peripheral blood. Next, to the extracted average fluorescence intensities for all of the hybrids identified in the tumor and in blood, we applied Z-score normalization to standardize and scale data, thereby aligning the staining intensity variations among Ab-oligos to a consistent scale. Then, we performed k-means clustering to the normalized fluorescence intensities for each Ab-oligo extracted from each hybrid cell to determine if hybrid cells clustered based on phenotype. We used an elbow method^40^ to determine the appropriate number of k-means clusters for our computational analysis. We displayed the normalized average fluorescence intensity of each antigen for each hybrid cell in a heatmap and grouped the cells by k-means cluster to visualize the phenotypes associated with each hybrid cluster. Using this analytical approach, we determined that the hybrids clustered into distinct subpopulations based on their protein expression profiles (Fig. 4), where tissue-associated hybrids harbored largely discrete phenotypes relative to their corresponding peripheral blood-associated hybrids. This finding was confirmed by performing pairwise Pearson’s correlations between subpopulations of hybrid cells in the tumor tissue to the subpopulations of hybrids in the peripheral blood of each patient. We found that the majority of hybrid cell subpopulations in peripheral blood were not represented in the tumor tissue, for example, in the CRC samples B2 was defined by higher expression of CD45, VWF, and pAKT and B7 was defined by high expression of CK8, αSMA, and Ki67 (Fig. 4A). In PDAC, B1 was defined by expression of CK8, VIM, and αSMA and B4 was defined by low expression of these markers (Fig. 4B). In both CRC and PDAC, some subpopulations were defined by higher CK8 expression (i.e. B7 in CRC and B1 in PDAC) and high CD45 expression (i.e. B2 in CRC and B3 in PDAC) (Fig. 4A,B ). Interestingly, in both CRC and PDAC samples, we identified hybrid subpopulations that were conserved across the tumor tissue and the peripheral blood, which were both driven by vimentin and αSMA expression, including T3, and B3 in the CRC samples and T6 and B2 in the PDAC samples (Fig. 4C).

## Discussion

Circulating cell-based biomarkers for cancer burden assessment are an increasingly important tool in cancer detection, treatment, and survival. Additionally, a scientific investigation aimed at developing biomarkers for monitoring disease burden and evolution, including assessing a patient’s risk for metastatic spread^15,17,18^, will yield important insights into the underlying biology that drives disease progression (e.g., neoplastic cell dissemination) and treatment evasion (e.g., cell signaling activation). However, in order to advance the field in these important areas, platforms for in-depth interrogation of single cell and subcellular properties and phenotypes, along with powerful imaging software to identify discrete subpopulations, are essential.

Hybrid cells (i.e., neoplastic cells that co-express tumor and immune proteins) represent a promising biomarker. Although relatively rare (< 1% of cells in blood and tumor) they are found in tumors (as tumor-associated hybrids) and peripheral blood (as circulating hybrid cells) of cancer patients. The Ab-oligo-based cyCIF platform described in this study^28–30^ offers the visual evaluation of these cells and their associated deep phenotyping. While there are a wide range of cyclic IF platforms available for analyzing tissue specimens, the majority of these approaches involve fluorophore bleaching^41–43^ or stripping^34,35,44^ for signal removal after each round, which makes them ill-suited for analyzing peripheral blood smears. PBMCs lack an extracellular matrix to withstand multiple rounds of chemical stripping, where cell loss and reduced antigenicity complicate their analyses^30^. Ab-oligo-based cyCIF relies upon UV light cleavage of the fluorescent probe from the antibody after each antibody staining round, resulting in minimal cell loss and cell damage^30^. Additionally, the Ab-oligo-based cyCIF platform is similar to mass spectrometry multiplexed workflows^45,46^, for example, CyTOF^47^ and MIBI^46^, in that all of the Ab-oligos are incubated onto the tissue in a single step, which reduces the overall workflow time. However, the Ab-oligo-based cyCIF approach is not hampered by the limited resolution, increased detection time, and low sensitivity inherent to those techniques. In addition, this platform is flexible in that the signal can be amplified for low abundance antigens or combined with traditional direct and indirect IF to detect a variety of biomarkers per sample^30^. Thus, these combined advantages were leveraged to evaluate hybrid cells from individual patients within their tumor tissues and compare them with those disseminated into peripheral blood.

Herein, we determined that hybrid cells detected in tumor and blood were heterogeneous and clustered into numerous subpopulations, based upon our discrete phenotyping panel to evaluate epithelial, immune, stromal, cell signaling, and functional characteristics, Table S1. Moreover, hybrid cells from the tumor tissue versus those in blood predominantly harbored distinct cellular phenotypes (including upregulated CD45 and CD44 (T1 CRC; T7 PDAC) and ECAD (T1 CRC; T1 PDAC); and discrete phenotype in peripheral blood (i.e., upregulated expression of CK8 B7 CRC and B1 PDAC), which may indicate that discrete properties are required for hybrid cell dissemination into peripheral blood. In addition, there were expected concordant phenotypes; most notably subpopulations that expressed increased levels of αSMA and Vimentin, known proteins associated with epithelial-to-mesenchymal transition (EMT)^48–52^. This later finding might indicate that hybrid cells within the tumor tissue that have increased EMT-associated proteins may have greater migratory capabilities and were thus retained among the neoplastic cells within the blood. While the identifiction of unique tumor-associated hybrid cell phenotypes were not detected in peripheral blood, which may indicate that either the small sampling of tumor tissue was not comprehensive enough to detect all tumor-hybrid cells, or that only phenotypically rare tumor-hybrid cells are functionally capable of dissemination. Of note, it is also feasible that some CHCs are not of tumor-origin, but rather from hybrid generation from epithelial cells within the tumor microenvironment. Most interesting were the distinct CHC phenotypes that could hint at unique biology for immune escape and survival in peripheral blood (i.e. increased CK8 in B7 CRC, B1 PDAC). Interestingly, enhanced levels of CK8 are associated with malignant transformation in head and neck squamous cell carcinoma^53,54^, and increased invasiveness in lung cancer^55^. Our observation may reveal the ability of disseminated tumor hybrid cells to be influenced by different microenvironments and thus alter their protein expression to survive. This paradigm is intriguing, as hybrid cells are likely to demonstrate increased plasticity given that they can be generated from fusion of two discrete cell lineages^15,16,56^. Overall, whether or not discrete protein expression profiles of hybrid cell subpopulations correlate with greater risk for metastatic spread of disease should be assessed in a larger cohort of patients that span the continuum of early to late stage cancer. However, the presence of enriched metastatic-associated phenotypes in hybrid cells that enter and survive in circulation indicates an enhanced potential for productive metastases. This seems especially feasible since CHCs have higher metastatic potential than CTCs^15,16^.

The coalescence of a biologically unique tumor cell population, with a gentle approach for its protein-based phenotyping and analyses by a powerful software platform, QiTissue, supports the expansion of biology and biomarker development. Further analyses of CHC subpopulations have the potential to unravel key signaling pathways for cell dissemination, immune evasion, and metastatic seeding. Downstream spatial analysis of unique hybrid cell subpopulations relative to discrete tumor structures may identify key information for cell extravasation from the primary tumor. On the clinical front, CHC phenotyping may lead to non-invasive assays that can identify a patient’s risk for metastasis or other important tumor insights that can aid in treating cancer patients and lead to increased survival. 

## Methods

### Human tissue and peripheral blood specimens

All peripheral blood and tumor tissue samples were collected and analyzed under approved protocols in accordance with the ethical requirements and regulations of the Oregon Health & Science University (OHSU) institutional review board (IRB). Informed consent was obtained from all subjects. Formalin-fixed paraffin-embedded (FFPE) tissue sections (5 µm) from normal tonsil, normal breast, breast cancer, colorectal cancer (CRC), and pancreatic ductal adenocarcinoma (PDAC) were obtained from the OHSU Knight Biolibrary. Patient matched CRC or PDAC tissue sections and peripheral blood specimens were collected under the purview of the IRB approved protocols for the Oregon Colorectal Cancer Registry and the Oregon Pancreatic Tumor Registry.

### Human peripheral blood preparation

Peripheral blood specimens were collected in heparin-coated vacutainers (BD Biosciences, CA) and processed the same day. PBMCs were isolated from whole blood using standard Ficoll-Paque PLUS (GE Healthcare, Chicago, IL, USA) density gradient protocols^16,57^. Briefly, whole blood was diluted with phosphate-buffered saline (PBS; 1.37 M NaCl, 27 mM KCl, 0.1 M Sodium phosphate dibasic, 18 mM KH2PO4, pH 7.4), layered over Ficoll then centrifuged. Isolated PBMCs were resuspended in PBS, 1.0 mM EDTA, and 2% FBS and applied to glass slides coated with poly-D-lysine (1 mg/mL, Fisher Scientific, PA), and incubated at 37 °C for 15 min to allow for their adherence to the slide. Processed PBMCs were then fixed with 4% paraformaldehyde (PFA, Sigma-Aldrich, St. Lois, MO) for 5 min, permeabilized with 0.5% Triton-X100 (Fisher Scientific, PA) for 10 min, fixed again with 4% PFA for 10 min, and dehydrated using graded ethanol baths. Slides were dried for 16 h at 25 ºC in a light restricted container and stored at 4 ºC.

### Mixed tumor cell line and PBMC slides

To validate Ab-oligo conjugates, mixed PBMC and tumor cell line slides were generated. Capan2 cells (ATCC, Manassas, VA) were grown in McCoy’s 5a Media (Thermo Fisher Scientific, Waltham, MA), and SW480 cells (ATCC, Manassas, VA) were expanded in Leibovitz’s L15 Media (Sigma Aldrich, St. Louis, MO) both with 10% fetal bovine serum (FBS) and in 5% CO_2_. Tumor cells were mixed in a 1:1 ratio with PBMCs isolated from healthy subjects and processed onto slides as described above.

### antibody-oligonucleotide conjugates (Ab-oligos)

Ab-oligos were generated as previously described^28,29^. Briefly, a unique 28 nucleotide (nt) single-stranded (ssDNA) docking strand (DS) was conjugated to the Fc region of each antibody via the SiteClick^™^ Antibody Azido Modification kit (ThermoFisher). The 26 nt complementary ssDNA IS carrying fluorophores and PCLs on each 5’ and 3’ end were used to detect the specific marker of interest. Alternatively^30^, 198 nt Amplification Strands (AmpS) with 28 nt complementarity to the DS and 10 repeated 15 nt segments complementary to 15 nt Amplification IS (Amp IS), each with one fluorophore and PCL on the 3’ end, were used for detection. All oligos were purchased from IDT (Coralville, IA). Antibody and oligo information is listed in Table S1.

### Immunofluorescence and cyclic immunofluorescence (cyCIF) tissue and blood specimen staining

To validate the Ab-oligos against indirect immunofluorescence staining, we used standard immunofluorescence protocols^28^. Briefly, FFPE tumor tissue sections were deparaffinized with xylene washes and then rehydrated with graded ethanol baths. Tissue was subjected to antigen retrieval by incubation in citrate buffer (pH 6; Sigma-Aldrich, St. Louis, MO) for 30 min at 100 °C, rinsed in 100 °C diH20, incubated in Tris–HCl buffer (pH 8, Thermo Fisher Scientific, Waltham, MA) for 10 min at 100 °C, then cooled to 25 °C. Prepared mixed cancer/PBMC slides were washed in PBS at 25 °C. For antibody staining, specimens were incubated with blocking buffer containing 2% bovine serum albumin (BSA, bioWORLD, Dublin, OH), 0.5% dextran sulfate (Sigma-Aldrich, St. Louis, MO), 0.5 mg/mL sheared salmon sperm DNA (Thermo Fisher Scientific) in PBS for 30 min at 25 °C. Comparison of specificity and sensitivity of the Ab-oligo conjugates within tissue and cells was assessed by comparing (1) indirect antibody staining (primary antibody + fluorescently-labeled secondary antibody), (2) Ab-oligo + fluorescently-labeled secondary antibody and (3) Ab-oligo + IS. Primary antibodies and Ab-oligos were diluted in the blocking buffer described above and incubated on tissue for 16 h at 4 °C. Blocking buffer containing 2% BSA, 0.5% dextran sulfate and 0.5 mg/mL sheared salmon sperm DNA in 2X Saline Sodium Citrate (SSC, VWR, Radnor, PA) was used to dilute secondary antibodies (see Table S1) to 1:500 and IS to 350 nM. Secondary antibodies and IS were incubated on samples for 45 min at 25 °C in the dark and samples were kept in the dark from this point on. Finally, they were counterstained with DAPI, cover-slipped with Fluoromount-G media (Thermo Fisher Scientific), and imaged using an AxioImager.M2 microscope (Carl Zeiss, Oberkochen, Baden-Wurttemberg Germany) and an AxioObserver with an Apotome3™ attachment and two cameras; a color MRm and an AxioCam 506 mono were used for widefield fluorescent imaging only. The system was driven by ZEN (3.4; Carl Zeiss). Following imaging of the specific biomarkers, samples were treated with UV light for 15 min as previously described^28^ to remove fluorescence signal. Samples were imaged in the same field of view (FOV) when possible, to demonstrate signal removal to the level of negative controls.

cyCIF staining was performed as previously described^29^. Briefly, samples were counterstained with DAPI and cover-slipped, and then imaged for a round 0 (R0) image of background fluorescence. The Ab-oligos were then incubated on the blood and FFPE tissue samples in one staining step. The unbound Ab-oligos were removed via washing and the Ab-oligos were post-fixed using 2% PFA for 10 min. Three to four ISs were added per round and imaged in spectrally distinct channels, DAPI (Zeiss 96 HE), Alexa Fluor 488 (AF488, Zeiss 38 HE), AF555 (Zeiss 43 HE), AF647 (Zeiss 50), and AF750 (Chroma 49007 ET Cy7). Following imaging, fluorescence signal was removed by UV treatment using a UV light box for 15 min, before the next round of distinct IS was added to the sample and repeated until all the biomarkers were imaged. Blood and FFPE tissues were digitally scanned with an AxioScan.Z1 microscope (Zeiss, Germany) equipped with a Colibri 7 light source (Zeiss). Exposure time was determined individually for each slide and antibody to ensure a good dynamic range without saturation. All images were taken with 20 × objective (Plan-Apochromat 0.8NA WD = 0.55, Zeiss) and stitching was performed using Zen Blue image acquisition software (Zeiss). One 5 µm tumor tissue section and corresponding patient-matched peripheral blood specimen (~ 1.5 mL) were analyzed per patient, per tumor type.

### Analyses

For Ab-oligo validation, Zen blue software (Carl Zeiss) and Fiji/ImageJ^58^ were used for image visualization. Images of indirect IF staining, Ab-oligo plus fluorescently labeled secondary antibody, Ab-oligo plus IS, Ab-oligo plus IS after UV cleavage, and corresponding negative controls were segmented in QiTissue or using Mesmer^59^. Mesmer nuclei segmentation was performed using a deep-learning based segmentation model^59^ with DAPI as the nuclear marker. Whole-cell segmentation was performed using ECAD as the cell membrane marker. Cytoplasmic segmentation masks were computed by subtracting nuclear segmentation masks from full cell body segmentation masks as previously shown^60^.

#### Signal to background ratio

Regions of interest (ROIs) with representative antibody staining were selected from Ab-oligo plus IS, Ab-oligo plus IS after UV cleavage, and IS only to calculate the signal-to-noise ratio and thus efficacy of Ab-oligo staining. For staining validation, ROIs contained representative cell numbers for tissue (176–3,247 cells) and blood (19 and 102 cells) for each specimen. For tissue, a single representative ROI that encompassed the majority of the tissue was used for validation, while n = 3 quadrants were analyzed to validate staining in peripheral blood. This difference was based on the fact that the tissue represented positive and negative cell populations to varying degrees, while the blood model was uniform. Cell fluorescence intensity values were extracted from data analyzed by QiTissue or using Mesmer^59^. For validation of Ab-oligos on tissue, the signal to background ratio was calculated by extracting the average whole cell fluorescence intensity for each positive cell in each representative ROI. Positive thresholding was based on normalizing to negative controls (IS Only or secondary antibody only) and for controls (IS Only and UV-Cleaved), cells with positive antibody staining were based on anticipated expression patterns within the tissue morphology. The average of the individual cell fluorescence values was calculated for each staining paradigm and the average intensity of each paradigm was then divided by the average intensity of the tissue with the IS only. For signal to background validation of Ab-oligos in the blood model, three representative ROIs were analyzed. Cells that were poorly segmented or negative cells whose segmentation overlapped with a positive cell’s expression were excluded from the analysis. For each cell, the average fluorescence intensity, cell cytoplasm, or cell nucleus was evaluated, depending upon the subcellular localization of the protein. Within each ROI, the cell fluorescence values for the positive cell population (i.e., cancer cell or PBMC) were averaged and divided by the average fluorescence of the negative cell population. Cancer cells were identified by size and morphology (i.e., they were notably larger than PBMCs). The “positive” cell population depended on the antibody being validated. Values for each ROI were averaged. For markers that stained both cancer cells and PBMCs (i.e., CD44 and αSMA), the signal to background ratio was calculated by normalizing the average intensity of the cells to the average of cells stained with the IS only. Signal to background ratio values were graphed using Prism (GraphPad Software, San Diego, CA).

For cyclic immunofluorescence, images from cyclic staining rounds were registered using QiTissue or a feature-based image registration^61^. The edges of the tissue sections and wells were excluded from analysis as well as areas with imaging artifacts (e.g., out of focus, bubbles). Using QiTissue, cells with high autofluorescence were removed from analysis based on the whole cell average fluorescence from the round 0 background channel. Hybrids were defined as expressing both CD45 and at least one of the markers in the epithelial cocktail: panCK, ECAD, or EpCAM. Positive expression was defined in QiTissue as a whole cell intensity average value above the positive staining threshold that was determined by normalizing to an unstained control. Average cell intensity values were extracted for each antigen in the phenotyping panel for each identified hybrid cell followed by Z-score normalization and k-means clustering which were performed using QiTissue. The number of k-means clusters was determined by the elbow analysis^40^ and heatmaps were created using Prism (GraphPad Software, San Diego, CA) where the hybrid cells were grouped based on k-means clusters.

Correlations between the hybrid clusters in tissue and peripheral blood were performed as previously described^60^. Briefly, a Pearson’s correlation was calculated using z-score normalized mean marker intensity for each hybrid cluster and graphed using the corrplot package in R Studio^62^.

### Supplementary Information

Supplementary Information.

## Data Availability

The datasets used and/or analyzed during the current study are available from the corresponding author on reasonable request.

## Abbreviations

αSMA: Alpha smooth muscle actin
Ab-oligo: Oligonucleotide conjugated antibody
Amp IS: Amplification imaging strand
AmpS: Amplification strands
CHC: Circulating hybrid cell
CK: Cytokeratin
CRC: Colorectal cancer
CTC: Circulating tumor cell
cyCIF: Cyclic immunofluorescence
CyTOF: Cytometry by time of flight
DNA: Deoxyribonucleic acid
DS: Docking strand
ECAD: E-cadherin
EGFR: Epidermal growth factor receptor
EMT: Epithelial to mesenchymal transition
EpCAM: Epithelial cell adhesion molecule
FFPE: Formalin-fixed paraffin-embedded
FOV: Field of view
IF: Immunofluorescence
IHC: Immunohistochemistry
IRB: Institutional review board
IS: Imaging strand
nt: Nucleotide
OHSU: Oregon Health & Science University
pAKT: Phosphorylated protein kinase, strain AK, thymoma (phosphorylated protein kinase B)
panCK: Pan-cytokeratin
PBMC: Peripheral blood mononuclear cell
PBS: Phosphate buffered saline
PCL: Photocleavable linker
PDAC: Pancreatic ductal adenocarcinoma
PFA: Paraformaldehyde
R0: Round 0
RNA: Ribonucleic acid
ROI: Region of interest
Sig:Bkgd: Signal to background ratio
ssDNA: Single stranded DNA
UV: Ultraviolet
VIM: Vimentin
VWF: Von Willebrand factor

## References

[1] Zhou H (2022). Liquid biopsy at the frontier of detection, prognosis and progression monitoring in colorectal cancer. Mol. Cancer.

[2] Kumar A (2023). Current and emerging therapeutic approaches for colorectal cancer: A comprehensive review. World J. Gastrointest. Surg..

[3] Groot VP (2018). Patterns, timing, and predictors of recurrence following pancreatectomy for pancreatic ductal adenocarcinoma. Ann. Surg..

[4] Pulumati A (2023). Technological advancements in cancer diagnostics: Improvements and limitations. Cancer Rep. (Hoboken).

[5] Sutton TL (2022). Circulating cells with macrophage-like characteristics in cancer: The importance of circulating neoplastic-immune hybrid cells in cancer. Cancers (Basel).

[6] Campos-da-Paz M (2018). Carcinoembryonic antigen (CEA) and hepatic metastasis in colorectal cancer: Update on biomarker for clinical and biotechnological approaches. Recent Pat. Biotechnol..

[7] Postel M (2018). Droplet-based digital PCR and next generation sequencing for monitoring circulating tumor DNA: A cancer diagnostic perspective. Expert Rev. Mol. Diagn..

[8] Castro-Giner F, Aceto N (2020). Tracking cancer progression: From circulating tumor cells to metastasis. Genome Med..

[9] Andree KC, van Dalum G, Terstappen LW (2016). Challenges in circulating tumor cell detection by the cell search system. Mol. Oncol..

[10] Millner LM, Linder MW, Valdes R (2013). Circulating tumor cells: A review of present methods and the need to identify heterogeneous phenotypes. Ann. Clin. Lab. Sci..

[11] Cohen SJ (2008). Relationship of circulating tumor cells to tumor response, progression-free survival, and overall survival in patients with metastatic colorectal cancer. J. Clin. Oncol..

[12] Miller MC, Doyle GV, Terstappen LW (2010). Significance of circulating tumor cells detected by the cell search system in patients with metastatic breast colorectal and prostate cancer. J. Oncol..

[13] Gallerani G (2023). Settling the uncertainty about unconventional circulating tumor cells: Epithelial-to-mesenchymal transition, cell fusion and trogocytosis. Int. Rev. Cell. Mol. Biol..

[14] Reduzzi C (2020). The curious phenomenon of dual-positive circulating cells: Longtime overlooked tumor cells. Semin. Cancer Biol..

[15] Gast CE (2018). Cell fusion potentiates tumor heterogeneity and reveals circulating hybrid cells that correlate with stage and survival. Sci. Adv..

[16] Dietz MS (2021). Relevance of circulating hybrid cells as a non-invasive biomarker for myriad solid tumors. Sci. Rep..

[17] Parappilly MS (2022). Circulating neoplastic-immune hybrid cells predict metastatic progression in uveal melanoma. Cancers (Basel).

[18] Walker BS (2021). Circulating hybrid cells: A novel liquid biomarker of treatment response in gastrointestinal cancers. Ann. Surg. Oncol..

[19] Zhang Y (2015). Patterns of circulating tumor cells identified by CEP8, CK and CD45 in pancreatic cancer. Int. J. Cancer.

[20] Lizier M (2016). Fusion between cancer cells and macrophages occurs in a murine model of spontaneous neu+ breast cancer without increasing its metastatic potential. Oncotarget.

[21] Clawson GA (2017). "Stealth dissemination" of macrophage-tumor cell fusions cultured from blood of patients with pancreatic ductal adenocarcinoma. PLoS One.

[22] Manjunath Y (2020). Circulating giant tumor-macrophage fusion cells are independent prognosticators in patients with NSCLC. J. Thorac. Oncol..

[23] Toyoshima K (2015). Analysis of circulating tumor cells derived from advanced gastric cancer. Int. J. Cancer.

[24] Lustberg MB (2014). Heterogeneous atypical cell populations are present in blood of metastatic breast cancer patients. Breast Cancer Res..

[25] de Wit S (2018). Classification of cells in CTC-enriched samples by advanced image analysis. Cancers (Basel).

[26] Takao M, Takeda K (2011). Enumeration, characterization, and collection of intact circulating tumor cells by cross contamination-free flow cytometry. Cytometry A.

[27] Liu Q, Liao Q, Zhao Y (2016). Myeloid-derived suppressor cells (MDSC) facilitate distant metastasis of malignancies by shielding circulating tumor cells (CTC) from immune surveillance. Med. Hypotheses.

[28] Jones JA (2021). Oligonucleotide conjugated antibody strategies for cyclic immunostaining. Sci. Rep..

[29] McMahon NP (2020). Oligonucleotide conjugated antibodies permit highly multiplexed immunofluorescence for future use in clinical histopathology. J. Biomed. Opt..

[30] McMahon NP (2023). Flexible cyclic immunofluorescence (cyCIF) using oligonucleotide barcoded antibodies. Cancers (Basel).

[31] Lin JR (2017). Multiplexed single-cell imaging: Past, present, and future. Assay Drug Dev. Technol..

[32] Ullal AV (2014). Cancer cell profiling by barcoding allows multiplexed protein analysis in fine-needle aspirates. Sci. Transl. Med..

[33] Decalf J, Albert ML, Ziai J (2019). New tools for pathology: A user's review of a highly multiplexed method for in situ analysis of protein and RNA expression in tissue. J. Pathol..

[34] Zrazhevskiy P, Gao X (2013). Quantum dot imaging platform for single-cell molecular profiling. Nat. Commun..

[35] Zrazhevskiy P, True LD, Gao X (2013). Multicolor multicycle molecular profiling with quantum dots for single-cell analysis. Nat. Protoc..

[36] Goltsev Y (2018). Deep profiling of mouse splenic architecture with CODEX multiplexed imaging. Cell.

[37] Wang Y (2017). Rapid sequential in situ multiplexing with dna exchange imaging in neuronal cells and tissues. Nano Lett..

[38] Saka SK (2019). Immuno-SABER enables highly multiplexed and amplified protein imaging in tissues. Nat. Biotechnol..

[39] Young Hwan C (2017). Deep learning based nucleus classification in pancreas histological images. Annu. Int. Conf. IEEE Eng. Med. Biol. Soc..

[40] Shi C (2021). A quantitative discriminant method of elbow point for the optimal number of clusters in clustering algorithm. EURASIP J. Wirel. Commun. Netw..

[41] Gerdes MJ (2013). Highly multiplexed single-cell analysis of formalin-fixed, paraffin-embedded cancer tissue. Proc. Natl. Acad. Sci. USA.

[42] Lin JR (2016). Cyclic immunofluorescence (CycIF), a highly multiplexed method for single-cell imaging. Curr. Protoc. Chem. Biol..

[43] Lin JR, Fallahi-Sichani M, Sorger PK (2015). Highly multiplexed imaging of single cells using a high-throughput cyclic immunofluorescence method. Nat. Commun..

[44] Stack EC (2014). Multiplexed immunohistochemistry, imaging, and quantitation: A review, with an assessment of Tyramide signal amplification, multispectral imaging and multiplex analysis. Methods.

[45] Angelo M (2014). Multiplexed ion beam imaging of human breast tumors. Nat. Med..

[46] Levenson RM, Borowsky AD, Angelo M (2015). Immunohistochemistry and mass spectrometry for highly multiplexed cellular molecular imaging. Lab. Invest..

[47] Giesen C (2014). Highly multiplexed imaging of tumor tissues with subcellular resolution by mass cytometry. Nat. Methods.

[48] Dauphin M (2013). Vimentin expression predicts the occurrence of metastases in non small cell lung carcinomas. Lung Cancer.

[49] Satelli A, Li S (2011). Vimentin in cancer and its potential as a molecular target for cancer therapy. Cell. Mol. Life Sci..

[50] Bogush TA (2020). A new approach to epithelial-mesenchymal transition diagnostics in epithelial tumors: Double immunofluorescent staining and flow cytometry. Biotechniques.

[51] Kalluri R, Weinberg RA (2009). The basics of epithelial-mesenchymal transition. J. Clin. Invest..

[52] Ding L (2014). alpha-Smooth muscle actin-positive myofibroblasts, in association with epithelial-mesenchymal transition and lymphogenesis, is a critical prognostic parameter in patients with oral tongue squamous cell carcinoma. J. Oral Pathol. Med..

[53] Gires O (2006). CK8 correlates with malignancy in leukoplakia and carcinomas of the head and neck. Biochem. Biophys. Res. Commun..

[54] Xu XC (1995). Increased expression of cytokeratins CK8 and CK19 is associated with head and neck carcinogenesis. Cancer Epidemiol. Biomark. Prev..

[55] Fukunaga Y (2002). Expression of cytokeratin 8 in lung cancer cell lines and measurement of serum cytokeratin 8 in lung cancer patients. Lung Cancer.

[56] Melzer C (2021). Spontaneous fusion of MSC with breast cancer cells can generate tumor dormancy. Int. J. Mol. Sci..

[57] Lin SJ (2002). Expression of adhesion molecules on T lymphocytes in young children and infants–a comparative study using whole blood lysis or density gradient separation. Clin. Lab. Haematol..

[58] Schneider CA, Rasband WS, Eliceiri KW (2012). NIH Image to ImageJ: 25 years of image analysis. Nat. Methods.

[59] Lowe DG, Lowe DG (1999). Object recognition from local scale-invariant features. Proceedings of the Seventh IEEE International Conference on Computer Vision.

[60] Burlingame EA (2021). Toward reproducible, scalable, and robust data analysis across multiplex tissue imaging platforms. Cell Rep. Methods.

[61] Greenwald NF (2022). Whole-cell segmentation of tissue images with human-level performance using large-scale data annotation and deep learning. Nat. Biotechnol..

[62] Wei, T. *R package 'corrplot': Visualization of a Correlation Matrix*. Vers. 0.92. https://github.com/taiyun/corrplot (2021).

