# Supplementary material for: Ultra high content analyses of circulating and tumor associated hybrid cells reveal phenotypic heterogeneity
Source: Sci Rep. 2024 Mar 28;14:7350. doi: 10.1038/s41598-024-57381-8 (PMC10973471; doi:10.1038/s41598-024-57381-8)
Supplement: Supplementary file 1 — Supplementary Information. [file 41598_2024_57381_MOESM1_ESM.docx]

**
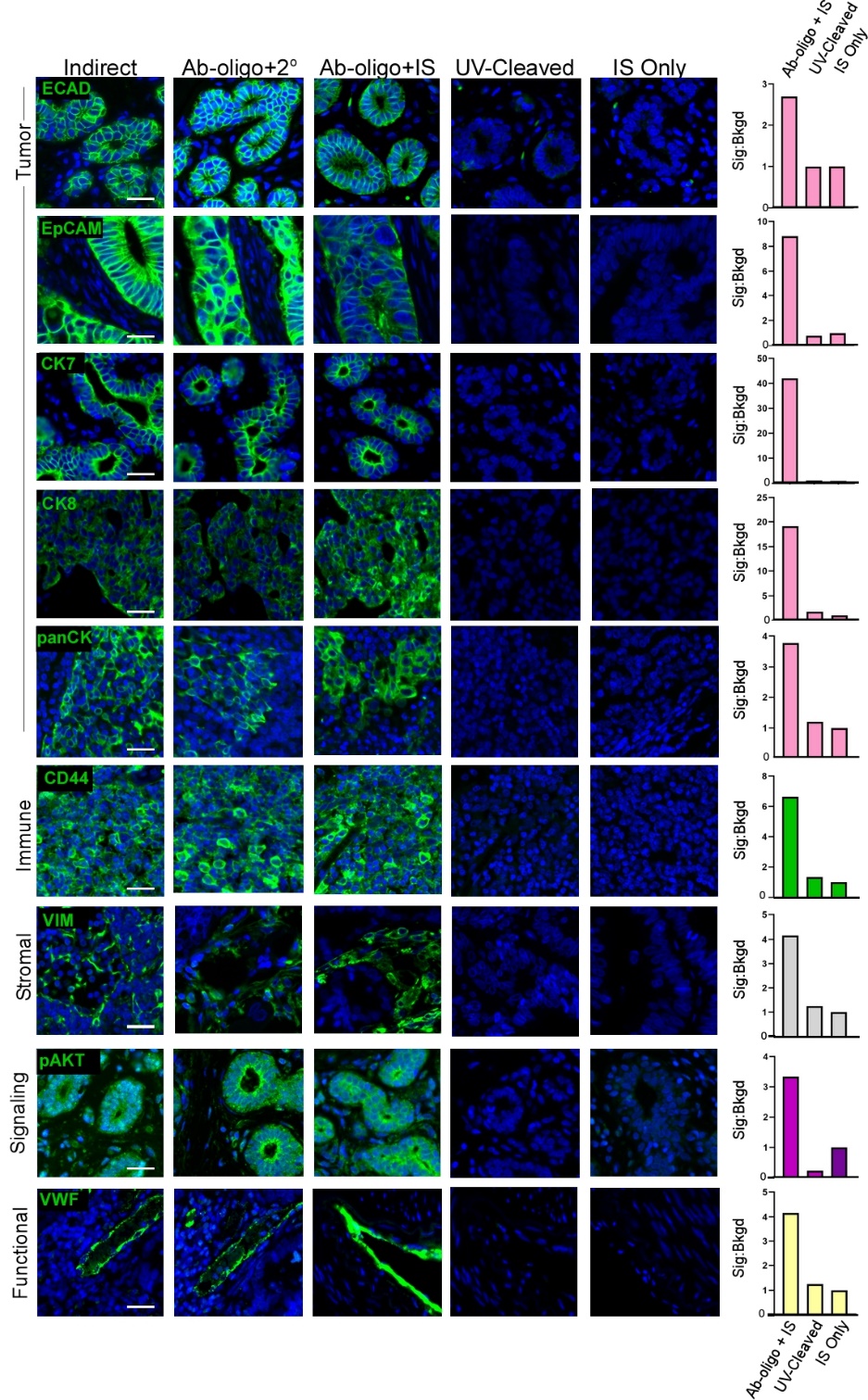
**

Figure S1. Validation of remaining Ab-oligo panel on formalin-fixed paraffin embedded (FFPE) 5 µm tissue sections. Antibody or control staining pattern (green) and DAPI nuclear stain (blue) shown in all panels. Antigen detection was by indirect immunofluorescence (primary + secondary antibody, 2°), Ab-oligo plus secondary antibody, and Ab-oligo plus imaging strand (IS). Control settings include UV-cleavage after Ab-oligo+IS staining, and IS only. Graphs depict signal to background ratio (Sig:Bkgd) of Ab-oligo+IS, UV-Cleaved, and IS Only controls normalized to the IS Only condition in stained tissue. Scale bar = 25 µm. Ab-oligo, oligo conjugated antibody; CK, cytokeratin; ECAD, E-cadherin; EGFR, epidermal growth factor receptor; EpCAM, epithelial cell adhesion molecule; IS, imaging strand; pAKT, phosphorylated protein kinase, strain AK, Thymoma (phosphorylated protein kinase B); panCK, pan-cytokeratin; Sig:Bkgd, signal to background ratio; UV, ultraviolet; VIM, vimentin ; VWF, von Willebrand factor.


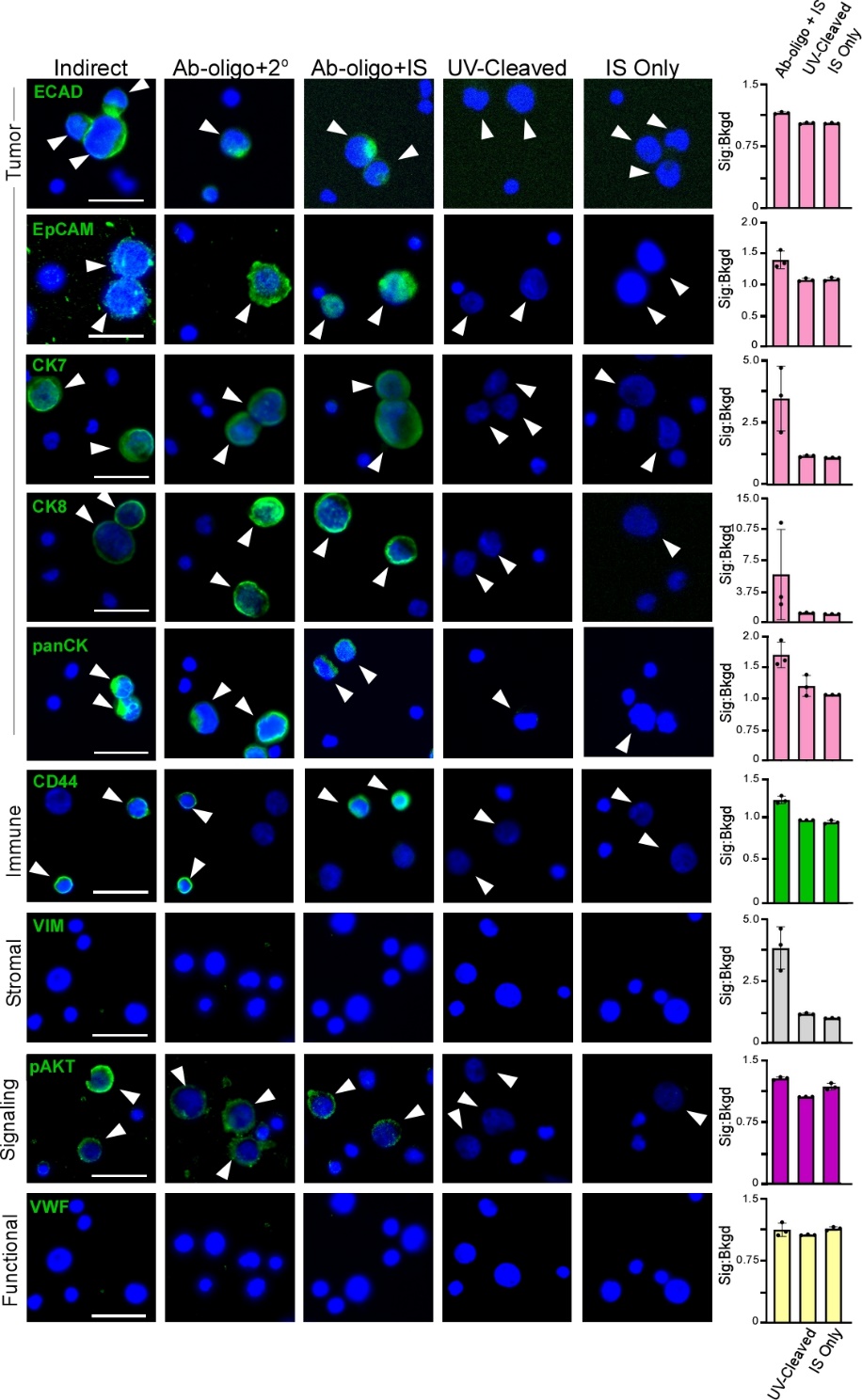


Figure S2. Validation of additional Ab-oligos on a model of peripheral blood (cancer cell line mixed with peripheral blood mononuclear cells, PBMCs). Antibody or control staining pattern (green) and DAPI nuclear stain (blue) shown in all panels. Antigen detection was by indirect immunofluorescence (primary + secondary antibody, 2°), Ab-oligo plus secondary antibody, and Ab-oligo plus imaging strand (IS). Control settings include UV-cleavage after Ab-oligo+IS staining, and IS only. White arrowheads designate staining on cells with antibody, or no staining on controls. Graphs depict signal to background ratio (Sig:Bkgd) of Ab-oligo+IS, UV-Cleaved, and IS Only controls normalized to the negative cell population within a staining paradigm (right). Scale bar = 20 µm. Ab-oligo, oligo conjugated antibody; CK, cytokeratin; ECAD, E-cadherin; EGFR, epidermal growth factor receptor; EpCAM, epithelial cell adhesion molecule; IS, imaging strand; pAKT, phosphorylated protein kinase, strain AK, Thymoma (phosphorylated protein kinase B); panCK, pan-cytokeratin; Sig:Bkgd, signal to background ratio; UV, ultraviolet; VIM, vimentin ; VWF, von Willebrand factor.

~~
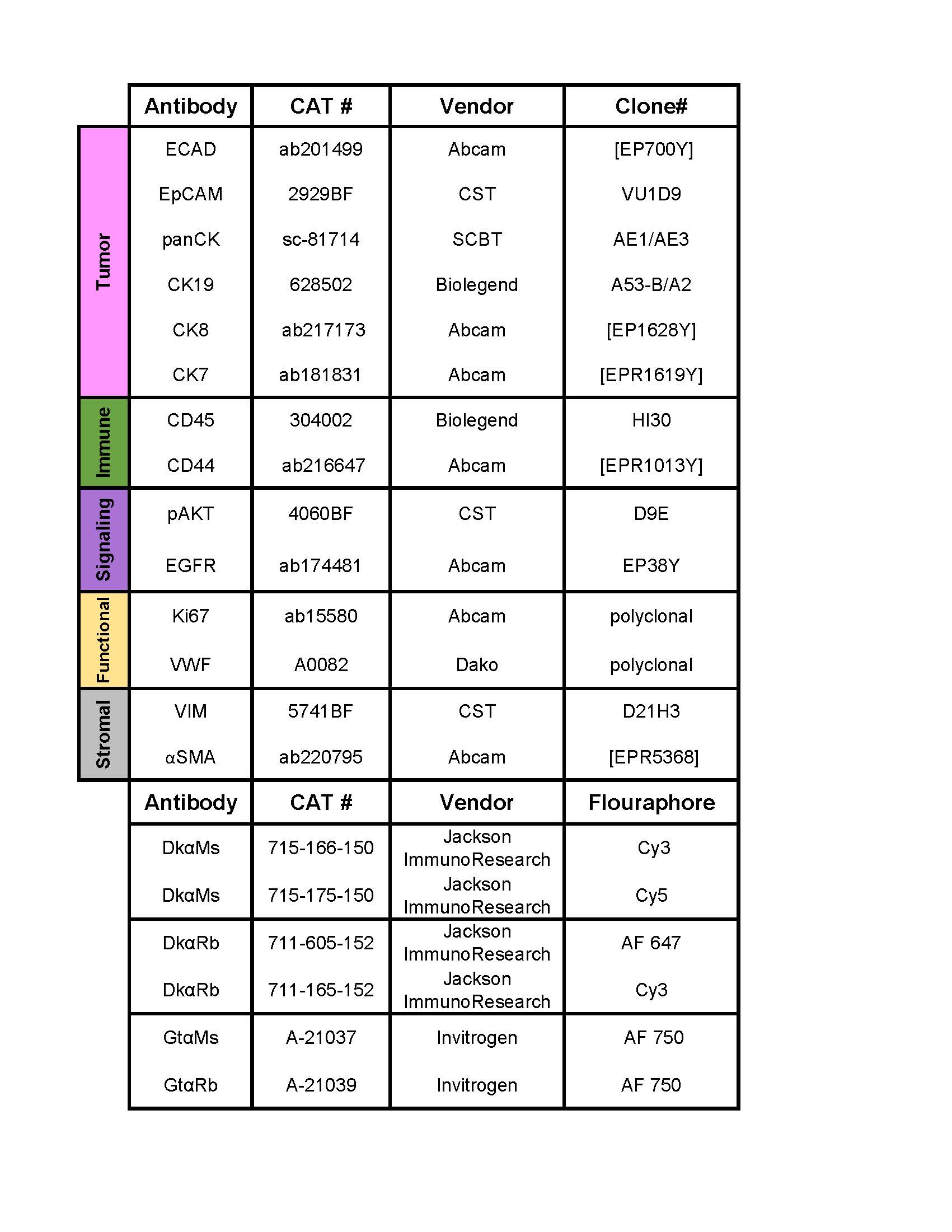
~~

Table S1. Antigens used in the phenotyping panel organized by category. The corresponding primary antibodies used for oligonucleotide conjugation resulting in Ab-oligos for validation. Secondary Antibodies used in validation listed below.
